# *Listeria monocytogenes*: Investigation of Fitness in Soil Does Not Support the Relevance of Ecotypes

**DOI:** 10.3389/fmicb.2022.917588

**Published:** 2022-06-13

**Authors:** Yann Sévellec, Eliette Ascencio, Pierre-Emmanuel Douarre, Benjamin Félix, Laurent Gal, Dominique Garmyn, Laurent Guillier, Pascal Piveteau, Sophie Roussel

**Affiliations:** ^1^Maisons-Alfort Laboratory for Food Safety, Salmonella and Listeria Unit, University of Paris-Est, French Agency for Food, Environmental and Occupational Health and Safety (ANSES), Maisons-Alfort, France; ^2^Agroecologie, AgroSup Dijon, INRAE, Bourgogne Franche-Comté University, Dijon, France; ^3^Risk Assessment Department, French Agency for Food, Environmental and Occupational Health and Safety (ANSES), University of Paris-Est, Maisons-Alfort, France; ^4^INRAE, UR OPAALE, Rennes, France

**Keywords:** *Listeria monocytogenes*, soil, fitness, survival, ecotype, strain origin, clonal complex, genome-wide-association study

## Abstract

*Listeria monocytogenes* (*Lm*) is a ubiquitous bacterium that causes the serious foodborne illness listeriosis. Although soil is a primary reservoir and a central habitat for *Lm*, little information is available on the genetic features underlying the fitness of *Lm* strains in this complex habitat. The aim of this study was to identify (i) correlations between the strains fitness in soil, their origin and their phylogenetic position (ii) identify genetic markers allowing *Lm* to survive in the soil. To this end, we assembled a balanced panel of 216 *Lm* strains isolated from three major ecological compartments (outdoor environment, animal hosts, and food) and from 33 clonal complexes occurring worldwide. The ability of the 216 strains to survive in soil was tested phenotypically. Hierarchical clustering identified three phenotypic groups according to the survival rate (SR): phenotype 1 “poor survivors” (SR < 2%), phenotype 2 “moderate survivors” (2% < SR < 5%) and phenotype 3 “good survivors” (SR > 5%). Survival in soil depended neither on strains’ origin nor on their phylogenetic position. Genome-wide-association studies demonstrated that a greater number of genes specifically associated with a good survival in soil was found in lineage II strains (57 genes) than in lineage I strains (28 genes). Soil fitness was mainly associated with variations in genes (i) coding membrane proteins, transcription regulators, and stress resistance genes in both lineages (ii) coding proteins related to motility and (iii) of the category “phage-related genes.” The cumulative effect of these small genomic variations resulted in significant increase of soil fitness.

## Introduction

*Listeria monocytogenes* (*Lm*) is a facultative intracellular pathogen responsible for listeriosis, a serious foodborne disease affecting both humans and animals. *Lm* is a ubiquitous bacterium, which can be found in many habitats including soil, water, plants, animals, foodstuffs and humans. Farm animals are important reservoirs of *Lm* and they contribute to the circulation of *Lm* in the farm environment and to its transfer in soil through fecal shedding (Hurtado et al., 2017). Increasing amounts of data on the prevalence of *Lm* in wildlife suggest that various species of wild animals can act as reservoirs for *Lm* and may participate in its transfer to soil (Yoshida et al., 2000; Hellstrom et al., 2008; Gismervik et al., 2015; Weindl et al., 2016; Hydeskov et al., 2019; Parsons et al., 2019). Other routes of transfer of *Lm* to soil include organic fertilization and decaying plant material (McLaughlin et al., 2011).

Since the first discovery of occurrence of *Lm* in soil samples collected from farms in 1960 (Welshimer, 1960), *Lm* has been considered as a telluric bacterial species. Follow-up studies, listed in Vivant et al. (2013) have confirmed the presence of *Lm* in different soil types including uncultivated and cultivated soils and meadows. Soil may play a pivotal role in transmission of *Lm* to cultivated plants and farm animals, eventually leading to contamination of foodstuffs (Piveteau et al., 2011; Vivant et al., 2013; Kallipolitis et al., 2020). However, a deep understanding of the ecology of *Lm* in soil and of the contribution of intrinsic factors and intraspecific diversity to its fitness in this habitat is required for the management of *Lm* in food systems from the natural environment to the food chain.

*Lm* is a genetically heterogeneous species divided into 13 serotypes and three major phylogenetic lineages, of which lineages I and II are the most frequently encountered (Orsi et al., 2011). These two lineages group the serotypes most commonly associated with human listeriosis, including serotypes 1/2b and 4b (lineage I) and serotype 1/2a (lineage II). Most strains are grouped into major clonal complexes (CCs) that have descended from a common ancestor and accumulated differences among themselves by a predominantly mutational process (Ragon et al., 2008; Chenal-Francisque et al., 2011; Cantinelli et al., 2013; Haase et al., 2014). CCs evolve slowly over large temporal and geographic scales (Chenal-Francisque et al., 2011; Cantinelli et al., 2013).

Hypervirulent and hypovirulent CCs have been identified (Maury et al., 2016). Hyper virulent CCs such as for instance CC1, CC2, CC6, are those most likely to cause disease, in particular central nervous system or maternal-neonatal listeriosis (Maury et al., 2016) and account for the majority of human listeriosis outbreaks, sporadic human cases (Painset et al., 2019), and animals listeriosis (Dreyer et al., 2016; Papić et al., 2019). On the contrary, hypo virulent CCs, such as for instance CC9 and CC121, merely cause disease in highly immuno-compromised patients and show limited virulence in humanized mouse models (Maury et al., 2016). Many typing studies have indicated that the relative prevalence of CCs in animals, human clinical cases and in food is strikingly different (Henri et al., 2016; Felix et al., 2018; Painset et al., 2019). This suggests that some *Lm* strains thrive better in specific ecological compartments. To date, the link between the genetic diversity of *Lm* (lineage, serotype and CCs), the strain origin (food, animal, and environment) and *Lm* ability to survive in soil has yet to be investigated. From the point of view of food safety, investigating the fate of hyper virulent strains in agri-food systems once transferred in soil is of particular interest. This could lead to innovative measures of control and risk assessment.

Genome-wide association studies (GWAS) represent today powerful tools for the identification of associations between genomic elements and phenotypic properties. GWAS is a top-down approach that involves testing large numbers of genetic variants in a population of individual organisms with a given phenotype. GWAS dealing with food-related phenotypes was recently applied to *Lm* traits associated to cold, salt, acid, desiccation stresses (Hingston et al., 2017; Fritsch et al., 2019) adhesion to polystyrene (Mahoney et al., 2022) and persistence in dairy farms (Castro et al., 2021).

In this study, GWAS was performed to elucidate the phenotype-genotype relationships in *Lm* soil survival. We focused on a dataset of 216 strains selected to be representative of the major CCs or sequence types (STs) circulating between three major compartments of *Lm*: natural and farming environment, wild/farm animals and food. This panel was implemented to investigate genomic determinants of soil survival following three working hypotheses (i) soil fitness depends on the strain origin (ii) soil fitness depends on the phylogenetic position of the strains and (iii) soil fitness can be associated with specific genes.

## Materials and Methods

### Strain Panel

The panel was composed of 216 *Lm* strains belonging to 33 clonal complexes from lineage I (*n* = 95) and lineage II (*n* = 121; Table 1 and Supplementary Table 1). The strains were collected over more than 30 years with most of them isolated after 2010, across 15 different European countries.

**TABLE 1:** Distribution of the 216 fully sequenced *Listeria monocytogenes* strains according to their origin, Lineage and clonal complex.

		Lineage I	Lineage II
Compartment	Sub-compartment	CC	CC
Food	Dairy product	CC1 (*n* = 4), CC4 (*n* = 4), CC6 (*n* = 4) CC2 (*n* = 1), CC217 (*n* = 1)	CC7 (*n* = 4), CC37 (*n* = 2), CC21 (*n* = 3)
	Fish product	CC2 (*n* = 5), CC6 (*n* = 3)	CC8 (*n* = 3), CC9 (*n* = 5), CC121 (*n* = 5), CC155 (*n* = 4)
	Meat product	CC1 (*n* = 4), CC2 (*n* = 4), CC6 (*n* = 4)	CC8 (*n* = 4), CC9 (*n* = 4), CC121 (*n* = 4), CC37 (*n* = 2)
	Vegetable and fruit product	CC1 (*n* = 3), CC2 (*n* = 4), CC6 (*n* = 6)	CC31 (*n* = 5), CC37 (*n* = 3), CC121 (*n* = 4)
Environment	Environment	CC1 (*n* = 4), CC2 (*n* = 2), CC4 (*n* = 5), CC5 (*n* = 1), CC6 (*n* = 3), CC54 (*n* = 1) CC77 (*n* = 1), CC220 (*n* = 1), ST663 (*n* = 1), ST666 (*n* = 1)	CC7 (*n* = 2), CC8 (*n* = 5), CC9 (*n* = 1), CC11 (*n* = 4), CC14 (*n* = 1), CC18 (*n* = 4), CC20 (*n* = 1), CC21 (*n* = 2), CC29 (*n* = 1), CC37 (*n* = 2), CC121 (*n* = 2), CC155 (*n* = 1), CC475 (*n* = 1), CC26 (*n* = 1)
Animal	Farm animal	CC1 (*n* = 3), CC2 (*n* = 1), CC5 (*n* = 2), CC6 (*n* = 1), CC54 (*n* = 2), CC59 (*n* = 1), CC77 (*n* = 2), CC220 (*n* = 1), CC224 (*n* = 3), CC315 (*n* = 2), CC379 (*n* = 1)	CC7 (*n* = 1), CC8 (*n* = 1), CC9 (*n* = 2), CC11 (*n* = 3), CC14 (*n* = 1), CC18 (*n* = 1), CC20 (*n* = 2), CC26 (*n* = 1), CC29 (*n* = 1), ST36 (*n* = 2), CC37 (*n* = 2), CC412 (*n* = 2)
	Wild animal	CC1 (*n* = 2), CC4 (*n* = 2), CC6 (*n* = 1) CC59 (*n* = 2), CC220 (*n* = 1), CC315 (*n* = 1)	CC7 (*n* = 2), CC8 (*n* = 1), CC9 (*n* = 1), CC11 (*n* = 2), CC14 (*n* = 3), CC18 (*n* = 1), CC21 (*n* = 3), CC26 (*n* = 1), CC29 (*n* = 1), CC31 (*n* = 1), CC37 (*n* = 1), CC121 (*n* = 2), CC412 (*n* = 1), CC415 (*n* = 1), ST184 (*n* = 1)
Total		95	121

The 97 strains isolated from the environment and primary farm production were part of the strain collection (Félix et al., 2022) built in the frame of the European project ‘‘LISTADAPT’’ (Adaptive traits of *Listeria monocytogenes* to its diverse ecological niches).^1^ In all, strains were selected from farm animals (bovine, ovine, caprine, and pigs), wild animals (deer, badger, slugs, dolphin, wild boar, fox, and roe deer) and soil. They were selected to be representative of the major CCs or STs found in the LISTADAPT collection (Félix et al., 2022). In order to maximize the genetic diversity, at least two to four strains were selected per CC and ST with the greatest geographical breadth and the highest degree of ST diversity within each CC (Supplementary Table 1).

Additionally, 99 strains were selected from four ready-to-eat (RTE) food categories according to the risk food matrixes given by Painset et al. (2019). Of these, 11 strains were provided by the European LISEQ collection (Painset et al., 2019) and the rest of food strains were extracted from the ANSES Maisons-Alfort Laboratory for Food Safety collection of food isolates. The 10 major CCs circulating in secondary and tertiary food production were previously determined from two previous French studies (Maury et al., 2016; Felix et al., 2018) and one conducted in Europe (Painset et al., 2019). At least four strains collected from four different European countries and from as many STs as possible were selected within CC in order to maximize the genetic diversity (Supplementary Table 1).

### Survival of Strains in Soil Microcosms

Soil was collected from the upper superficial tillage layer at a depth of 0–10 cm in a field located in an experimental farm unit of INRAE (Bretenière, France). It is a silty clay soil containing 38% clay, 56% silt and 6% sand, with a pH of 6.9. Soil was sieved to 4 mm within 24 h after sampling then stored at –20°C for less than 1 month until use. Soil microcosms were prepared in 24-well microtiter plates by adding 0.5 g (equivalent dry weight) of soil per well. Plates were incubated 24 h at 25°C prior inoculation. Inocula were prepared from frozen stocks, by culturing in 10 mL of Trypton Soy Broth for 24 h at 25°C, then by sub-culturing (1%v/v) for 16 h under the same incubation conditions. Cells were washed twice in sterile distilled water then the final cell density was adjusted in sterile distilled water in order to inoculate microcosms at 10^6^ CFU/g dry soil. The total volume of inoculum was calculated in order to adjust the soil water content to 60% of its Water Holding Capacity. Inoculated microcosms were incubated 36 h at 25°C. After incubation, 5 mL of Tryptone salt were added per soil microcosms. Soil slurries were serially diluted and *Lm* populations were numerated on Rapid’L mono (incubation 24–48 h at 37°C). The absence of indigenous *Lm* in soil was checked by direct plating on Rapid’L mono before inoculation. Experiments were performed in triplicates.

### Correlation Between Strain Origin and Soil Fitness

The strains were clustered based on their survival rate using the *hclust* R function (version 3.6.1) with the complete linkage method for hierarchical clustering. Ordinal logistic regression was used to explore the link between the strain ability to survive in soil and its origin. The *polr* R function was used to carry out the ordinal logistic regression.

### Genome Sequencing

The sequenced genomes were produced using paired-end sequencing (2 × 150 bp) on different Illumina instruments (NovaSeq 6000/NextSeq 500/Illumina HiSeq 2500). The raw reads were processed using the harmonized in-house workflow ARTWork.^2^ This pipeline performs various analyses (*de novo* assembly, scaffolding, annotation, quality control, inter-and intra-species contamination) that have been described in detail in previous studies (Vila Nova et al., 2019; Palma et al., 2020). Genome with a mean coverage < 30× and scaffolds with a length <200 bp were excluded. A cut-off for assembly was set at 200 contigs and an assembly length outside 2.7–3.2 Mbp. Sequence type (ST) and CC were also predicted based on the Listeria Multilocus ST (MLST) scheme (Moura et al., 2016). The accession numbers, the associated metadata and the metrics of the genome assemblies are provided in Supplementary Table 1.

### Phylogenomic Reconstruction of the Population Structure

Prediction of SNPs-Indels was performed using the Ivarcall2 pipeline (Felten et al., 2017) for alignment of the reads against the reference genome of Lm EGD-e (NC_003210.1). The phylogenetic tree was calculated on the pseudogenome based on genomic distance using iqtree V.1.6.9 (Schmidt et al., 2014). The best-fitted model for this dataset was determined to be a three-substitution type model with equal base parameter, empirical base frequencies (Kimura, 1981) and allowed proportion of invariant site (K3P + F + I). The tree was corrected for homologous recombination events using ClonalFrame (Didelot and Wilson, 2015). The genome of the *L. innocua* strain FR-FAR-WT-170 was used as outgroup to root the tree. The final recombination aware tree was visualized using iTOL (Letunic and Bork, 2019).

### K-mer Based Genome-Wide-Association-Study

First, variable length K-mers were counted on all genomes using fsm-lite software with default parameters.^3^ The GWAS was then performed on the strain survival rate treated as a continuous trait using pyseer software (V.1.3.4) K-mer association study was applied on the full dataset (216 genomes) and three subsets including genomes from lineage I (*n* = 95), lineage II (*n* = 121) and genomes belonging to CC6 (*n* = 22).

Briefly, ClonalFrame inference (phylogeny tree) was used to generate a strain similarity matrix, based on the random effect model (phylogeny_distance.py script). The significance of the associations was determined according to their Bonferroni corrected *p*-value using the script count_pattern.py provided in the pyseer package: (i) full dataset *p*-value < 3.09–08 (ii) Lineage I *p*-value < 1.92–07 (iii) Lineage II *p*-value < 9.84–08) (iv) CC6 *p*-value < 9.00–03. K-mers significantly associated with soil fitness were then annotated on the reference genomes presenting the highest number of k-mers: LV-BOV_CP_29 for the whole collection (22/24) and the lineage II (2,148/2,242), DE-RDE-FE-17 for the lineage I (74/162) and FR-FI-U-UN-418 for the CC6 (2,243/3,056) (annotate_hits_pyseer script). The average maximum allele frequency (MAF) was determined by pyseer for each genes identified (Supplementary Table 2). The gene function was confirmed by BLAST analysis on the NCBI database.^4^ The PHASTER web server was used for the rapid identification of prophage sequences^5^ (Arndt et al., 2016, 2019).

### Correlation Between Phylogeny and Soil Fitness

In order to assess the association of soil phenotypes along the branches of the phylogenetic tree, the Bayesian inference method (Ansari and Didelot, 2016) was applied. The R package TreeBreaker was used in order to infer the evolution of a discrete phenotype distribution across a phylogenetic tree and to divide the tree into segments where their distributions are constant (Ansari and Didelot, 2016). Therefore, the core genome excluding recombination events and the discrete phenotypic traits (“fast” = 0, “slow” = 1) were supplied as input to this program.

## Results

### Survival Rate of 216 Lm Strains in Soil

**FIGURE 1:**
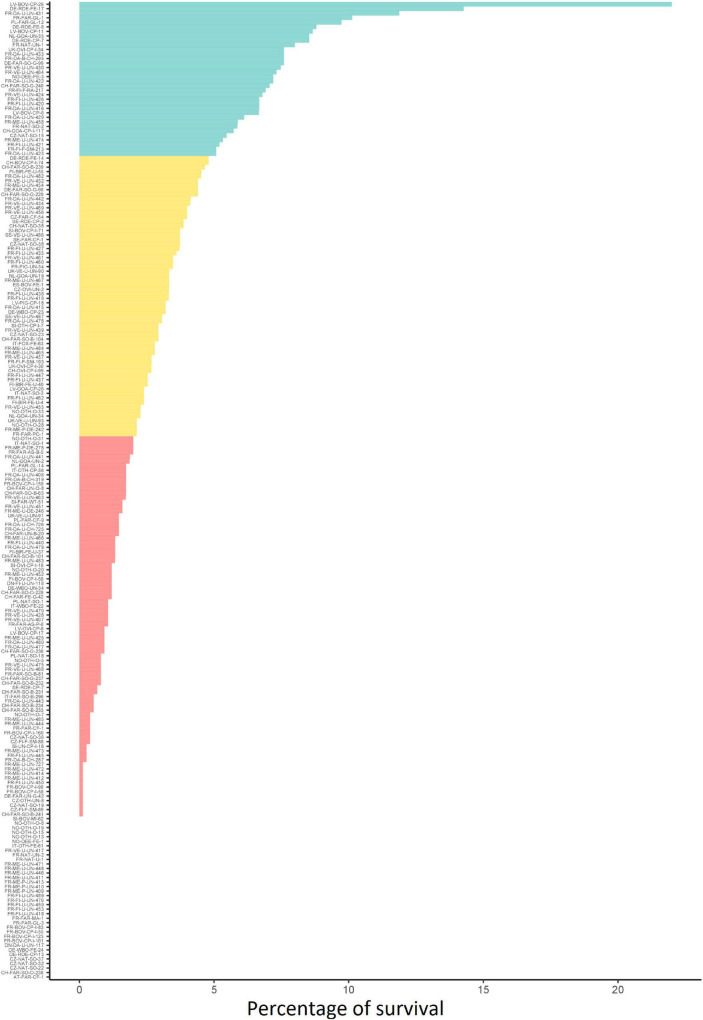
Soil survival phenotype of 216 strains tested. The three phenotypic classes of strain ability to survive in soil (blue = “good,” orange = “moderate” and red = “poor”) were determined by ascending hierarchical clustering.

The fate of the 216 strains was investigated during incubation in soil. The results were strain-dependent. Survival ranged from zero to 22% (Figure 1 and Supplementary Table 1). Hierarchical clustering grouped the strains into three discrete phenotypes, i.e., “poor,” “moderate,” and “good” according to their percent of survival in soil (Figure 1). For 54.4% of the strains, less than 2% of the initial population was detected after 36 h of incubation in soil (“poor survivors” = phenotype 1) and 36 strains had a survival rate of zero. Of these 36 strains, 12 were isolated from animal, 15 from food and 9 from environment. Thirty percent of the strains had survival rates between 2 and 5%. (“moderate survivors” = phenotype 2). A minority of strains (15.6%) had survival rates over 5% (“good survivors” = phenotype 3). The strain LV-BOV-CP-29 (CC11, Lineage II) isolated from animal presented the highest survival rate in soil.

**FIGURE 2:**
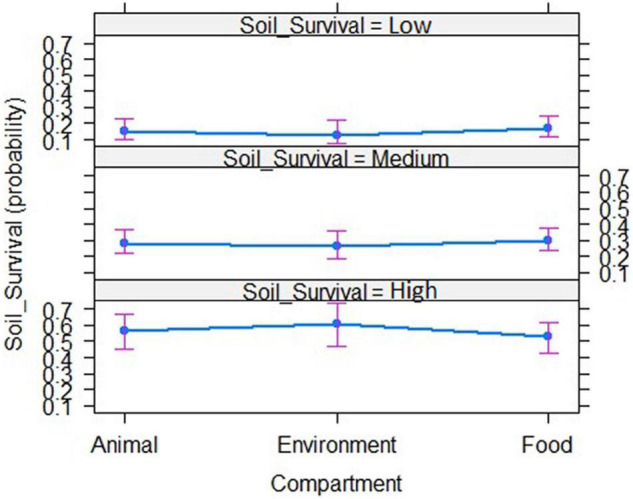
Ordinal logistic regression to assess correlations between the strain’s origin and its soil fitness from the phenotypic data collected from the 216 strains.

Ordinal logistic regression of the phenotypes observed of the 216 strains was performed in order to investigate possible correlations between the phenotype and the origin of the strains. No significant correlation between the three ordered phenotypes characterizing strain’s potential to survive in soil and strain’s origin could be evidenced (Figure 2). Survival in soil does not depend of strain’s origin.

### Correlation Between Phylogeny and Soil Fitness

**FIGURE 3:**
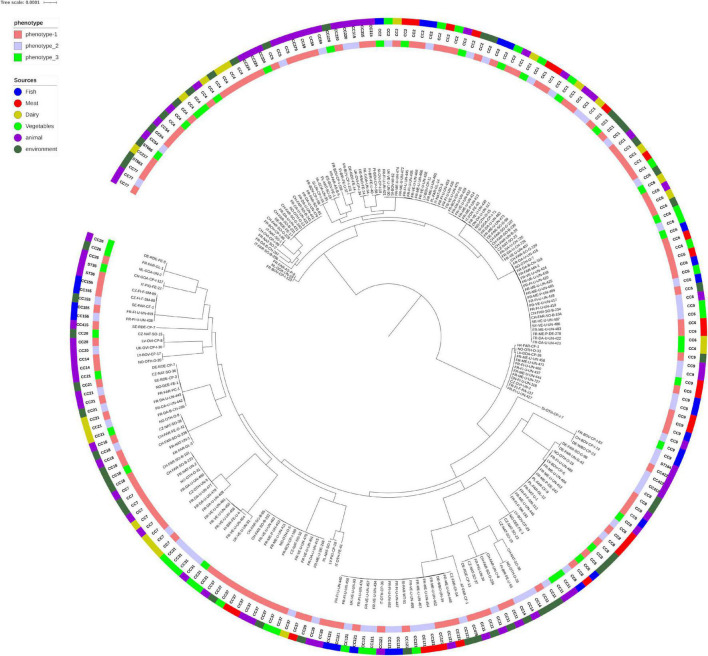
Phylogeny of the 216 *Listeria monocytogenes* genomes. The inner band indicates the phenotype of the strains and the outer band the different compartments and sub-compartments. The genomes clustered in two main branches corresponding to the lineage I and II. Inside each lineage, the strains were clustered according to their CC except for strains from CC14 (6 strains) and CC11 (9 strains) which present a polyphyletic structure between ST14 and ST91 and between ST 451 and ST11, respectively, (Figure 1). The pan-genome consisted of 2,384 core genes (plus a soft core of 126 genes present in more than 95% of the genomes).

The distribution of the observed phenotypes within the phylogenetic tree based on the core genomes is presented in Figure 3. The three classes of phenotypes are randomly distributed on the tree leaves and analysis using Bayesian inference showed no stead. In addition, the posterior probabilities of phenotype changes on given branches (corresponding to Lineages, CC or any other clades) are not significant (Supplementary Figure 1). Survival in soil dos not depend of the phylogenetic position of strains.

### Genome-Wide Association Study

The details of each gene and the number of significant k-mers associated with soil fitness are available in Supplementary Table 2.

#### Genes Associated With Soil Fitness in the Full Dataset (216 Genomes)

**FIGURE 4:**
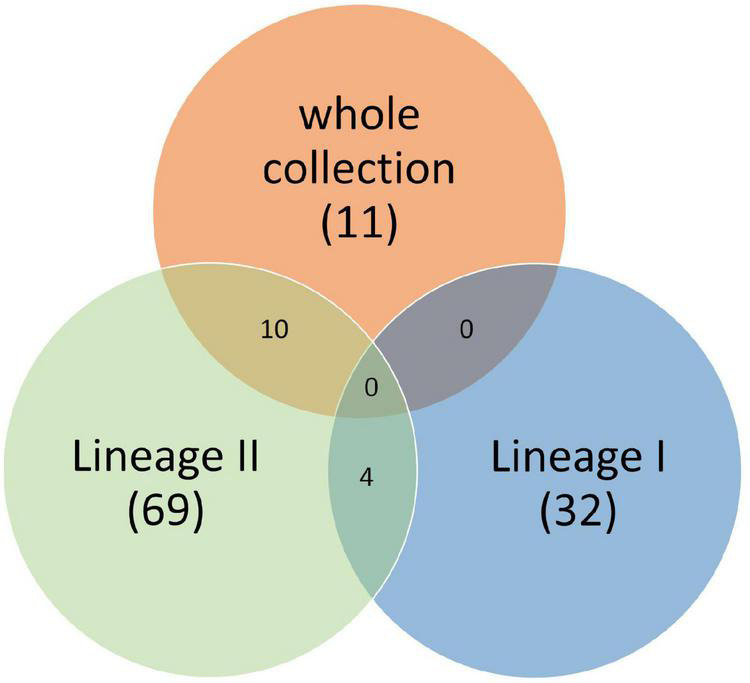
Venn diagram of genes significantly associated with soil survival extracted from the whole set of genomes, from the subset of Lineage I and from the subset of Lineage II.

Twenty-four k-mers, located in 11 genes, were associated with soil fitness (Figure 4 and Supplementary Table 2) with twenty two k-mers detected in the genome of strain LV-BOV-CP-29 (Strain presenting the highest survival rate 22% survival rate, lineage II, CC11). These 11 genes were present in few genomes (between three and six depending on the k-mer) with an average maximum allele frequency (MAF) between 0.014 and 0.0279. Interestingly, these genes were significantly associated with soil fitness specifically in Lineage II strains (Figure 4 and Supplementary Table 2).

Among the 11 genes identified, nine were significantly associated with high soil survival: the *whiB* gene, detected only in the best soil survivor strain (LV-BOV-CP-29) coding for a transcriptional regulator (Zheng et al., 2012) and eight genes annotated as prophage elements (Supplementary Table 2). Blast analysis of the LV-BOV-CP-29 genome revealed that these eight genes belong to a 38 Kb prophage integrated in the tRNA-Arg locus which shared 91% identity and 79% coverage with the listeria phage LP-101 (NC_024387.1) (Denes et al., 2014).

Additionally, two genes were significantly associated with poor soil survival: The *rpoC* gene codes for the beta subunit of the DNA-directed RNA polymerase and has been linked to resistance to cephalosporin and the *HCFLCJGM_00555* gene encodes a hypothetical protein (Supplementary Table 2).

#### Genes Associated With Soil Fitness in Lineage I Strains (95 Genomes)

**FIGURE 5:**
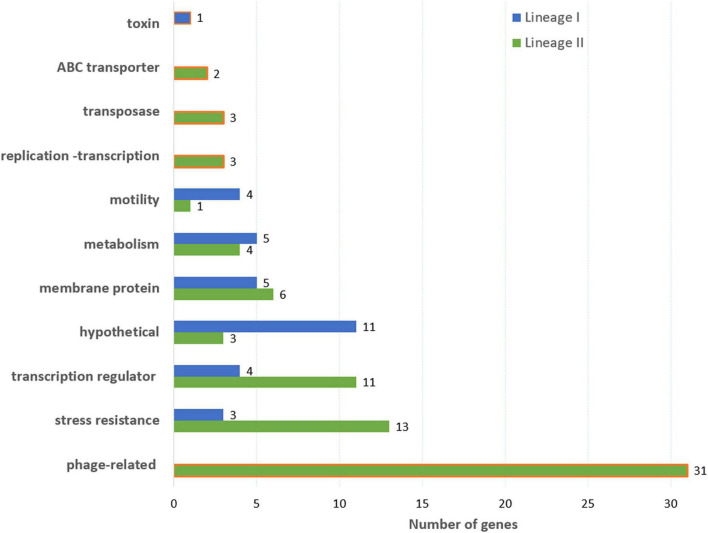
Function of genes associated with soil fitness in lineage I (95 genomes) and lineage II (121 genomes). The orange frame indicates that the gene function is only found in one lineage.

In lineage I, 162 k-mers covering 32 genes and presenting a low average MAF (between 0.02 and 0.07) were significantly associated with soil fitness (Figure 5 and Supplementary Table 2). Out of the 32 genes, 11 encode hypothetical proteins, four are transcription regulators including two regulators of stress response (PerR and SrlR), four genes are linked to *Listeria monocytogenes* motility (Figure 5 and Supplementary Table 2). Five genes coding membrane proteins were also identified, including four internalins (inlA, inlJ and two putative internalines) and two ABC transporters. Five genes belong to metabolism: Two of them are linked to resistance to oxidation (*msrA* and *yqhD*), two to cyanoamino acid metabolism (*gmuD_1* and *gmuD_2*) and one related to fructose metabolism (*malL_2*). Finally, significant variation was identified in a putative anti-bacterial toxin gene. These 32 genes were not identified in the full dataset of 216 genomes (Figure 5).

For four genes (*msrA*, *IEOCHNDG_01235*, *gpx1* and *mall_2*) 88 k-mers detected were found in the genome of strains presenting poor soil survival. The 28 remaining genes were associated with high soil survival (Supplementary Table 2).

#### Genes Associated With Soil Fitness in Lineage II (121 Genomes)

In lineage II, 2,243 k-mers covering 63 genes and 6 intergenic regions (Figure 5) were significantly associated with soil fitness. Most of these genes presented a low MAF (between 0.017 and 0.5 with only six genes presenting a MAF value above 0.1). 29 genes coding for phage elements (38 kb phage of LV-BOV-CP-29), 13 regulation factors (including three linked to stress resistance: *perR*, *srlR*, and *ctsR*), 12 stress resistance genes, 4 membrane proteins and the *ackA* gene associated with motility were detected. Notably, four of these genes (*perR*, *cwlK*, *lytC*, *nagA*) code for proteins involved in membrane metabolism or organization, especially in connection with growth promotion and stress induced membrane rearrangement.

The 69 genes detected for the lineage II include all the genes identified on the full dataset except *HCFLCJGM_01110* (Figure 5) and four genes, *srlR* (coding a gluticol operon repressor), *perR* (coding a protein associated with peroxide resistance), *iap* (coding a secreted endopeptidase) and *ycaD* (coding a putative MFS transporter) also detected in lineage I.

For 57 genes, 95% of the k-mers identified were linked to high soil survival. For 12 genes, 94 k-mers were associated with poor soil survival. Among them, 43 k-mers were found in the gene *HCFLCJGM_00555* and the intergenic region upstream this gene as well as 20 k-mers located in the *msmE* gene coding for an ABC transporter (Supplementary Table 2).

#### Genes Associated With Poor Soil Fitness in CC6 (Lineage I) (22 Genomes)

3,056 k-mers were identified as significantly associated with soil fitness in strains from CC6. These k-mers were located in 41 genes and 1 intergenic region (Supplementary Table 2). The majority of k-mers (2,310/3,056) were located in seven genes. *Prokka_01766* is annotated as a pseudo gene corresponding to an HTH domain containing protein (706 k-mers). The *ycaD* gene is a putative MFS-transporter and a major salt resistance facilitator (457 k-mers). *Prokka_01773* was annotated as a hypothetical protein (449 k-mers). *ActA* (189 k-mers) is coding a virulence factor. *YlxH* codes a flagellum site determining protein (172 k-mers). *NemR* codes a transcriptional regulator of the bile resistance efflux pump (170 k-mers). Finally, three genes (*Prokka_01769*, *Prokka_01773* and *Prokka_01774* encodes putative internalinprotein (81, 420, and 167 k-mers, respectively).

Most of the k-mers (2,242/3,056) were linked to poor soil survival. Seventeen prophage related genes were associated with poor soil fitness, of which five were related to phage LP-030-3 (NC_024384.1) (Supplementary Table 2). These five genes were detected in seven CC6 strains with poor soil survival and were also identified in other CCs that presented poor soil fitness such as CC7 strains (6/7 strains) and CC121 strains (11/18 strains).

## Discussion

Because little information is currently available on the fate of the various Clonal Complexes under conditions relevant to outdoor environments, we designed experiments on a panel of 216 strains representative of the overall genetic diversity and sources of *Lm* (natural environments, farm environments, wildlife, farm animals, food). The most prevalent CCs detected in an aggregated collection of outdoor environment strains were included in the panel of strains (Felix et al., 2018; Painset et al., 2019; Papić et al., 2019). Similarly, strains representative of the genetic diversity of *Lm* found in food were selected among the 10 most prevalent CCs found in 4 different food categories (Painset et al., 2019). To our knowledge, this is the first in-depth comparative investigation of a comprehensive collection of *Lm* strains grouping a balanced number of food and outdoor environment strains.

Survival in soil was used as proxy for the fitness of *Lm* in outdoor environments. Soil is a complex and heterogeneous environment in which bacteria may encounter a range of harsh conditions. Upon its arrival in soil, *Lm* has to face competition for resources and antibiosis depending on the characteristics and diversity of the soil microbiome (McLaughlin et al., 2011; Vivant et al., 2013; Bruce et al., 2017; Smith et al., 2018; Spor et al., 2020) and prophage infection (Williamson et al., 2007; Srinivasiah et al., 2008). Similarly, abiotic stress (nutrient starvation, temperature, osmotic variations and heavy metal exposure) may occur in soil (Oliver, 2010; Smith et al., 2018). Soil fitness is likely to require the contribution of a large array of genomic factors as suggested by deep transcriptome variations upon arrival of *Lm* in soil (Piveteau et al., 2011; Vivant et al., 2015).

In this strain panel, “poor” soil survivors were present in every CC, and no CC was strongly associated with “good” soil fitness. Information on the variability of *Lm* fitness in soil is scarce. However, a previous study suggested that survival in soil is strain-dependent (McLaughlin et al., 2011). In a longitudinal survey of farms and natural environments in North America, one genotype (Lineage II-Ribotype DUP-1039C) was significantly associated with both natural environments and farms (Sauders et al., 2006). This ribotype actually groups CC9, CC14, CC204, CC368, and CC436. However, in our study, the strains from CC9 and CC14 did not present higher survival rates in soil than strains from the other CCs of the dataset.

The panel of 216 genomes representative of *Lm* strains found in soil, animals and food was analyzed in order to obtain insight into the genetic determinants of *Lm* fitness in soil. We used genome-wide-association-studies in order to identify genomic markers linked to a phenotype. These studies using linear mixed models are a powerful tool to capture small variations underlying phenotypic differences (San et al., 2020). In the present work, genomic features significantly associated with soil fitness were identified through association studies. Only 11 genes were associated to the soil fitness of the full dataset of 216 genomes Our results confirm that soil fitness requires the contribution of several functions, and suggest that minor gene variations confer extra advantage when facing biotic and abiotic edaphic challenges. Strikingly, the number of gene variants found to be associated with survival in soil was greater in Lineage II strains (69) than in Lineage I strains (32). This is intriguing and calls for further investigations taking into account the evolutionary history of both Lineages.

While not detected in the dataset of 216 genomes, three transcription regulators associated with stress response were detected in both Lineages and were associated mostly with high soil fitness with only 6 hits out of 119 associated with poor soil fitness. The *perR* gene codes a putative peroxidase stress response regulator involved in H_2_O_2_ resistance and cell growth (Rea et al., 2005). The closely located *ycaD* gene, also linked to soil fitness in CC6 strains, codes a putative MFS transporter. This gene is known to be a major facilitator of salt resistance (Duché et al., 2002). Finally, *srlR* encodes a putative glucitol operon regulator involved in stress response as previously demonstrated for *Lactobacillus paracasei* (Palud et al., 2018). The variants of these genes were, however, different between Lineage I and II. This emphasizes how different genomic variations in key genes can induce similar effects.

In Lineage I and II alike, most of the variants connected to coping with environmental stresses were associated with transcription regulation, stress response, membrane protein and motility. As a feature of a ubiquitous bacterial species, a large percentage of the genome of *Lm* codes regulators (Glaser et al., 2001; den Bakker et al., 2013; Vivant et al., 2013). Response of *Lm* to the edaphic environmental factors requires transcription regulation and fine-tuning of gene expression (Piveteau et al., 2011; Vivant et al., 2015). In Lineage II strains, “good” soil fitness was linked to variations in the sequence of 11 transcription factors coding genes. Among these, *ctsR* encodes the class 3 stress gene repressor of *Lm* (Nair et al., 2000; Karatzas et al., 2003) and *ackA* is involved in chemotaxis and motility (Gueriri et al., 2008).

In Lineage I, *flaA* coding a flagelin and *cheV* involved in chemotaxis (Karatan et al., 2001) were associated with soil fitness. Likewise, in CC6 strains, multiple variants of *ylxH*, coding FlhF the protein involved in flagelin localization, were associated with high (46 k-mer) and poor (126 k-mer) soil fitness. The *ctsR* gene involved in flagellar transcription was associated with soil fitness in lineage II and in CC6 strains. Motility and chemotaxis associated genes are known to contribute to stress resistance in *Listeria* (Casey et al., 2014; Vivant et al., 2017) and are up-regulated in soil as a strategy to cope with harsh environments (Vivant et al., 2017).

Stress response is another important feature of the saprophytic lifestyle of *Lm* and the capacity to trigger the general stress response is required for survival in soil (Brunhede et al., 2020; Marinho et al., 2020). Our findings further support the importance of stress response in soil fitness. Interestingly, 13 stress resistance-coding genes in lineage II and five in lineage I were predicted to affect soil fitness. These genes include resistance to oxidative stress (*cysK*, *uvrC* in Lineage II; *msrA* and *yqhD* in Lineage I) (Duché et al., 2002; Hain et al., 2012; Liu et al., 2019; Patange et al., 2019) and resistance to antimicrobial agents (*folP* or *rpoC*) (Braschi et al., 2018; Wang et al., 2019). GWAS also highlighted significant associations with genes related to cell division and shape of the bacterial envelop. Altogether, these results show that the characteristics of the surface of the bacterium are a key feature of fitness in the edaphic environment, as differential transcriptome analysis suggested previously (Piveteau et al., 2011; Vivant et al., 2017).

GWAS underlined variations in genes coding internalins and uncharacterized membrane proteins as well. Two Internalin genes, inlJ and inlA were associated to high soil survival in lineages I,II and CC6 while three putative internalins were linked to poor soil fitness in the CC6. Some internalins such as inlL and inlA are involved in adherence and biofilm formation (Popowska et al., 2017) while the capacity to form biofilms improves soil survival (Salazar et al., 2013). Therefore, biofilm formation could be a decisive factor in soil survivability.

This study highlighted several phage-related genomic regions. In Lineage II strains, variations in the region of bacteriophage LP-101 correlated to soil fitness. Several genes from this phage region were detected, in particular a variant of *yqbO*. In *Bacillus subtilis* this gene codes a component of a defective prophage known to provide protection against other prophages (Klumpp and Loessner, 2013; Suzuki et al., 2020). It is likely that this prophage plays an important role in soil survival of Lineage II strains. On the contrary, 17 prophage-related genes presented variations specifically associated with “poor” soil fitness in CC6 strains. Most of them were associated with LP-030-3, a prophage integrated in *comK*. In *Bacillus subtilis comK* codes the master regulator required for expression of late competence genes. In *Listeria monocytogenes*, functional ComK is involved in stress regulation and phagosomal escape (Chatterjee et al., 2006; Rabinovich et al., 2012). This region is considered a rapid adaptation island because the mosaic structure of the *comK* prophage region enables rapid recombination (Verghese et al., 2011). In particular, excision of prophage A118 from the c*omK* region restores expression of *ComK*, without entering the lytic phase (Rabinovich et al., 2012). Unfortunately, to date investigations on the role of LP-030-3-like prophages at this locus are lacking.

In this study, a panel of strains was built to cover the genomic diversity of *Lm* found in natural environments, animals and food. GWAS was performed on this panel in order to investigate the genomic features associated with the fitness of *Lm* in soil. Not surprisingly, fitness in this complex heterogeneous environment depends on the performance of a combination of several cellular processes. The synergy between several genomic variations could enhance the overall fitness of *Lm* clones among genetically related strains as well as between strains that are more distant. Several genomic variants of genes related to cell surface, transcription regulation and stress response were identified as significantly associated with soil fitness in our genetically diverse panel of *Lm* strains. This investigation also highlighted the importance of prophages in soil fitness but effects were either beneficial or detrimental according to the nature of the phage.

To gain a better understanding of the contributing effects of each of the genetic factors identified in this study, it would be of interest to investigate a larger genomic panel. It would then be possible to test the predictive power of these factors possibly by applying machine learning. The more promising factors could then be investigated by transcriptome analysis and reverse genetics.

## Data Availability Statement

The datasets presented in this study can be found in online repositories. The names of the repository/repositories and accession number(s) can be found in the article/Supplementary Material.

## Author Contributions

YS contributed substantially to the study design, acquisition of strains and the corresponding genomes, data analysis, quality control of the genomic dataset as well as writing and editing the manuscript. EA was in charge of all to the phenotypic experiments and contributed to writing and revision of the manuscript. P-ED contributed substantially to the data analysis, as well as writing and editing the manuscript. BF contributed to the acquisition and selection of strains. LGa and DG contributed to the design and analysis of the phenotypic experiments. LGu was in charge of statistical analysis, selection of strains for sequencing and helped in drafting the manuscript. PP participated in the study design, provided strains from soil and designed and coordinated the study, and contributed to writing and editing of the manuscript. SR designed and coordinated the study and contributed to writing and editing of the manuscript. All authors read and approved the final manuscript.

## Conflict of Interest

The authors declare that the research was conducted in the absence of any commercial or financial relationships that could be construed as a potential conflict of interest.

## Publisher’s Note

All claims expressed in this article are solely those of the authors and do not necessarily represent those of their affiliated organizations, or those of the publisher, the editors and the reviewers. Any product that may be evaluated in this article, or claim that may be made by its manufacturer, is not guaranteed or endorsed by the publisher.

## Footnotes

1
https://onehealthejp.eu/jrp-listadapt/

2
https://github.com/afelten-Anses/ARtWORK

3
https://github.com/nvalimak/fsm-lite

4
https://www.ncbi.nlm.nih.gov/

5
https://phaster.ca/

## Supplementary Material

Supplementary Figure 1 Evolution of a discrete soil phenotype distribution blue dot = “good,” orange dot = “moderate” and red dot = “poor”) on a phylogenetic tree including the genomes of 216 *Listeria monocytogenes* strains. The thickness of the branches is proportional to the posterior probability of having a change point. No change point was identified by treeBreaker. This shows that the three classes of phenotypes are randomly distributed on the tree leaves. The Bayesian inference method showed no steadiness distribution of phenotypes.

Supplementary Table 1 Characteristics of the strain dataset.
