# Supplementary figures and images for: Listeria monocytogenes: Investigation of Fitness in Soil Does Not Support the Relevance of Ecotypes
Source: Front Microbiol. 2022 Jun 13;13:917588. doi: 10.3389/fmicb.2022.917588 (PMC9234652; doi:10.3389/fmicb.2022.917588)

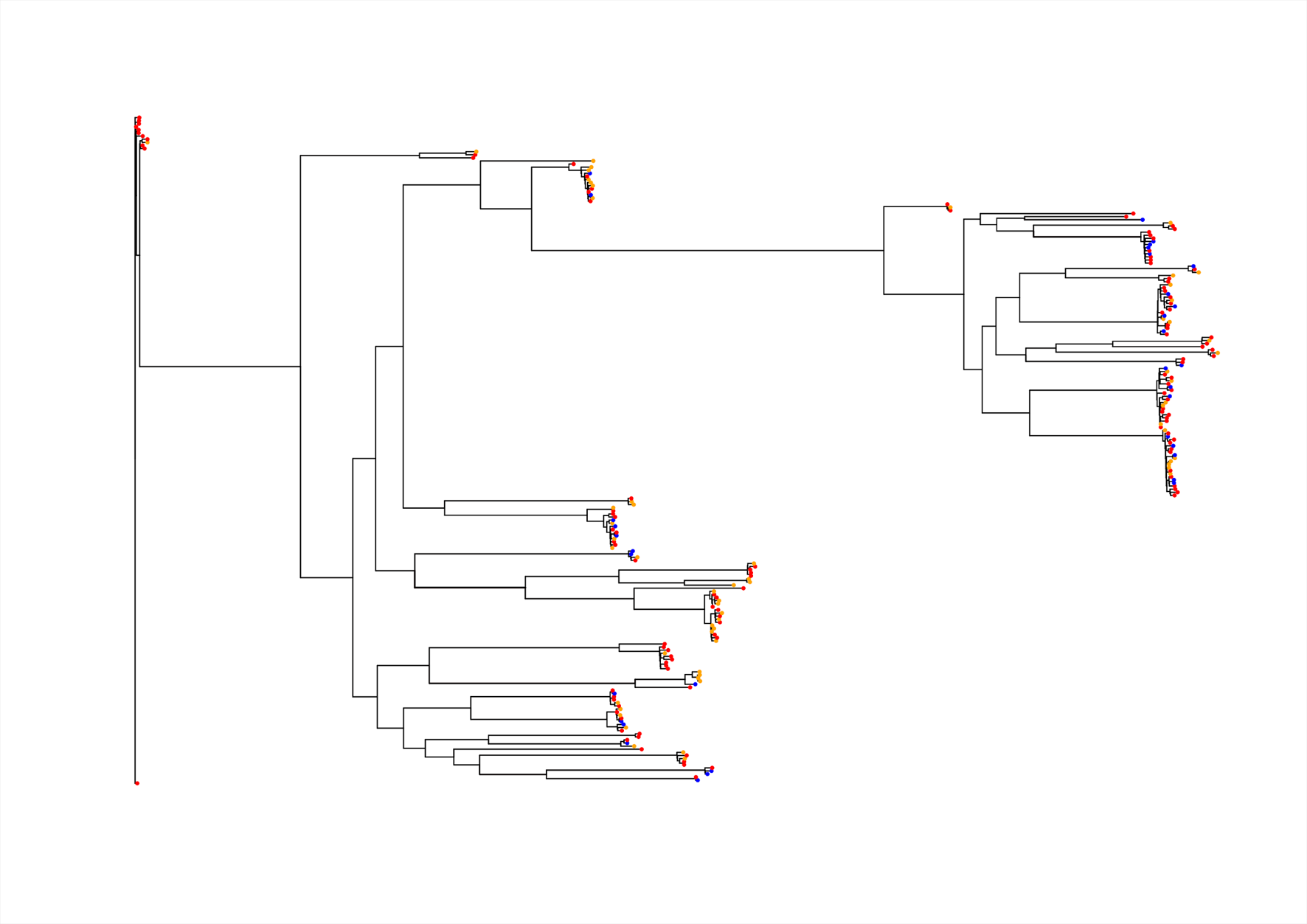

Supplement: Supplementary Figure 1 — Evolution of a discrete soil phenotype distribution blue dot = “good,” orange dot = “moderate” and red dot = “poor”) on a phylogenetic tree including the genomes of 216 Listeria monocytogenes strains. The thickness of the branches is proportional to the posterior probability of having a change point. No change point was identified by treeBreaker. This shows that the three classes of phenotypes are randomly distributed on the tree leaves. The Bayesian inference method showed no steadiness distribution of phenotypes. [file Image_1.TIFF]
